# Current training on the basics of robotic surgery in the Netherlands: Time for a multidisciplinary approach?

**DOI:** 10.1007/s00464-016-4970-2

**Published:** 2016-05-18

**Authors:** Willem Brinkman, Isabel de Angst, Henk Schreuder, Barbara Schout, Werner Draaisma, Lisanne Verweij, Ad Hendrikx, Henk van der Poel

**Affiliations:** 1 Department of Urology, Erasmus MC, Rotterdam, The Netherlands; 2 UMC Utrecht Cancer Center, Department of Gynaecologic Oncology, University Medical Centre Utrecht, Utrecht, The Netherlands; 3 Department of Urology, Alrijne Hospital, Leiden, The Netherlands; 4 Department of Surgery, Meander Medical Centre, Amersfoort, The Netherlands; 5 The Netherlands Institute of Health Services Research (NIVEL), Utrecht, The Netherlands; 6 Department of Urology, Catharina Hospital, Eindhoven, The Netherlands; 7 Department of Urology, Netherlands Cancer Institute, Amsterdam, The Netherlands

**Keywords:** Training, Robot, Surgery, Urology, Gynecology, Simulation

## Abstract

**Introduction:**

The following research questions were answered: (1) What are the training pathways followed by the current robot professionals? (2) Are there any differences between the surgical specialties in robot training and robot use? (3) What is their opinion about multidisciplinary basic skills training?

**Methods:**

An online questionnaire was sent to 91 robot professionals in The Netherlands. The questionnaire contained 21 multiple-choice questions focusing on demographics, received robot training, and their opinion on basic skills training in robotic surgery.

**Results:**

The response rate was 62 % (*n* = 56): 13 general surgeons, 16 gynecologists, and 27 urologists. The urologists performed significantly more robotic procedures than surgeons and gynecologists. The kind of training of all professionals varied from a training program by Intuitive Surgical, master-apprenticeship with or without duo console, fellowship, and self-designed training programs. The training did neither differ significantly among the different specialties nor the year of starting robotic surgery. Majority of respondents favor an obliged training program including an examination for the basics of robot skills training.

**Conclusion:**

Training of the current robot professionals is mostly dependent on local circumstances and the manufacturer of the robot system. Training is independent of the year of start with robotic surgery and speciality. To guarantee the quality of future training of residents and fellows in robot-assisted surgery, clear training goals should be formulated and implemented. Since this study shows that current training of different specialities does not differ, training in robotic surgery could be started by a multidisciplinary basic skills training and assessment.

**Electronic supplementary material:**

The online version of this article (doi:10.1007/s00464-016-4970-2) contains supplementary material, which is available to authorized users.

Currently, an increasing number of professionals across several specialties are using robot-assisted laparoscopy to perform surgery [1–3]. The transition from traditional open and laparoscopic surgery to this advanced technology makes procedures different and involves lack of haptic feedback, remote surgical control, and stereoscopic vision compared to laparoscopic surgery or open surgery. The pioneers in robot-assisted laparoscopy encountered new difficulties: master-apprenticeship learning was impossible due to the lack of experienced colleagues. Also in robotic surgery, if a supervisor is available, only verbal guidance instead of hands-on assistance can be given, due to the single console, which allows for only one operator. Consequently, simulation training was advocated from the start of robot-assisted laparoscopy. The manufacturer of the da Vinci Surgical System, currently the only approved system for robot-assisted laparoscopy, provided a mandatory start-up training program for all new customers. Previous studies report the effectiveness of various simulation-based training tasks for robot surgery, which will shorten the learning curve and improve the technical and nontechnical difficulties of robot-assisted laparoscopy [4, 5].

While the first generation of professionals had to learn robot-assisted laparoscopy without a supervisor, there is now a shift to a new generation that has the possible advantage of a supervisor in their hospital. Nevertheless, since these new users are not new customers, the training program provided by the manufacturer is not mandatory. Therefore, a gap now occurs for residents or fellows, who have not received basic training in robot-assisted surgery. This possible gap is equal for the different specialties using the robot. To prevent training by doing surgery directly on patients, and since basic robot training could be equal for the different specialties such as general surgery, gynecology, and urology, a multidisciplinary basic robotic skills training could be a feasible and effective training method. Before such a program can be developed, it is important to first investigate the training pathways of the current robot users, differences between specialities and the opinions of users about multidisciplinary basic skills training.

In this study, we aim to answer the following research questions: (1) What are the training pathways followed by the current robot surgeons? (2) Are there any differences between the specialties in robot training and robot use? (3) What is their opinion about multidisciplinary basic skills training?

## Method and materials

For this nationwide, multidisciplinary study, we developed a specific questionnaire in order to answer our research questions. The questionnaire was online based using Surveymonkey^®^ software. The questionnaire was sent to all known surgical robot users in The Netherlands, identified by their national society. In total, 91 medical specialists were invited to participate in this study. After the initial invitation, one reminder was sent.

### Questionnaire

The questionnaire contained 21 multiple-choice questions. In the first part, demographic variables such as the year of start with robot-assisted laparoscopy and the number of procedures were investigated. In the second part, questions specifically focused on robot training, e.g., what kind of training they had received, how many hours they had spent on training, and how many procedures they had performed under supervision. The final part of the questionnaire focused on their opinion on a basic skills training in robotic surgery.

### Data analysis

Statistical Package for the Social Sciences (SPSS) version 21 was used for the analyses. To analyze the differences between the specialties, we used cross tabs with Chi-square test and correlation was calculated with the Spearman’s rho. The alpha level was set at 0.05.

## Results

Of 91 invited specialists, 56 completed the questionnaire resulting in an overall response rate of 62 % (surgeons 65 %, gynecologists 59 %, urologists 61 %). One respondent did not complete the questionnaire and was excluded from the study. Of all included participants, 13 were general surgeons, 16 were gynecologists, and 27 were urologists. The median age was 48 years (range 36–61). Of the respondents, 50 were male and 6 were female.

**Fig. 1:**
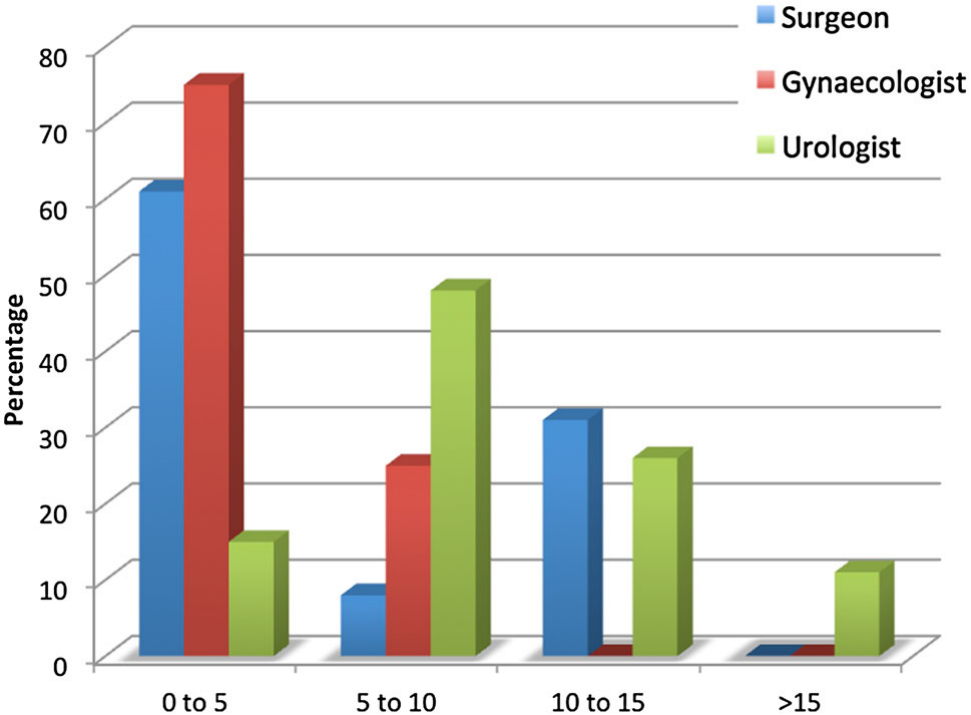
Number of procedures per month (in percentage)

All respondents had previous laparoscopic experience prior to the start of robotic surgery except for one. The year the specialists had begun performing robotic surgery varied from the year 2000–2014 (surgeons 2000–2014, gynecologist 2006–2013, and urologist 2002–2014) and did not differ significantly between the specialties. According to this questionnaire, urologists performed significantly more robotic procedures than surgeons and gynecologists, and surgeons performed more procedures than gynecologists. (Figure 1) (Chi-square test, *p* value = 0.001).

### Training experience

**Fig. 2:**
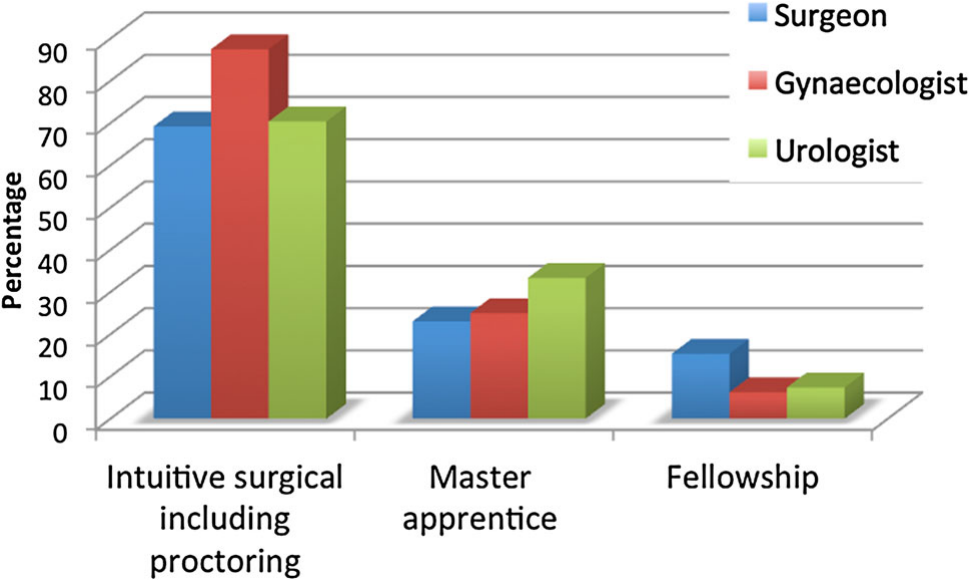
Type of training experienced

**Fig. 3:**
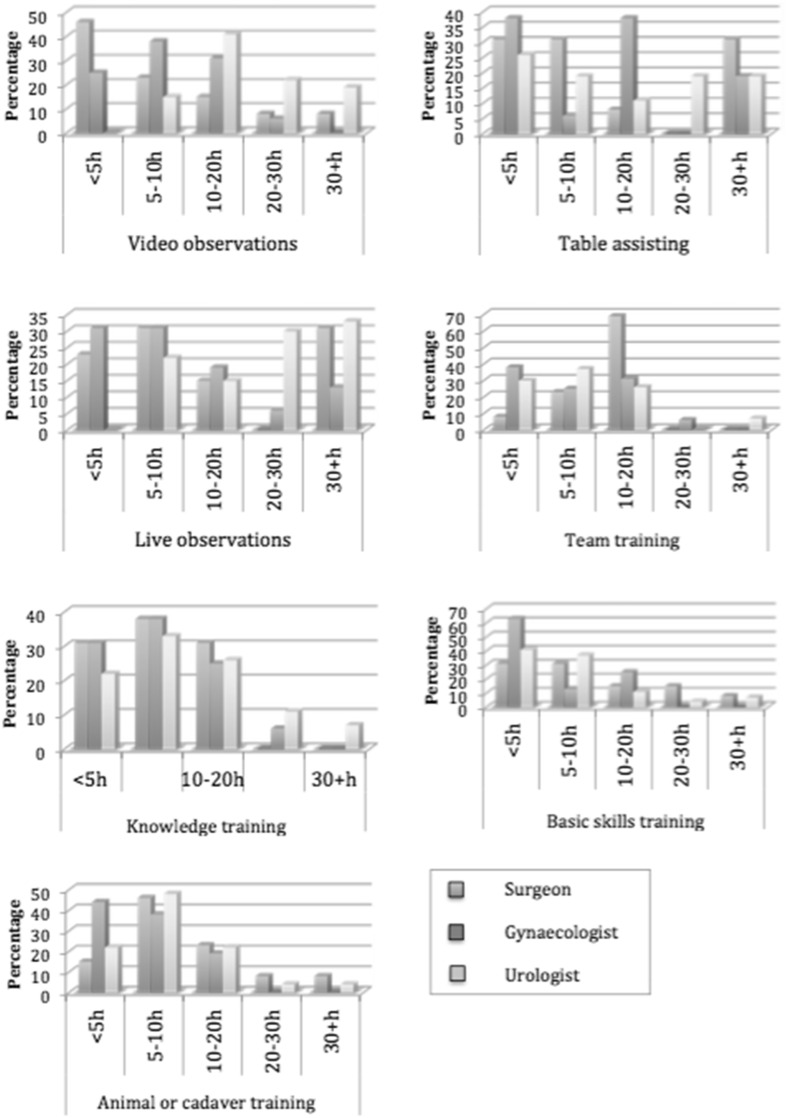
Hours spent on different training types

All participants had previous laparoscopic experience prior to the start of robotic surgery except for one. The sorts of training varied from a training program by Intuitive Surgical, master-apprenticeship with or without duo console, fellowship, and self-designed training programs (Fig. 2). The hours spent on the different sorts of training are shown in Fig. 3. The training did neither differ significantly among the different specialties nor the year of start.

According to the questionnaire, the surgeons were supervised during a median of 5 (0–55), the gynecologists during a median of 6 (0–55), and urologists during a median of 10 procedures (2–75). This difference between the different specialties was not significant. Only 10.7 % of the participants used a duo console for supervision. Again, no significant differences between specialties existed (Pearson Chi-square test: 6.247; *p* value = 0.620). The number of supervised procedures was correlated neither to year of start with robot-assisted laparoscopy nor to age in years (Spearman’s rho = −0.13; −0.22).

**Fig. 4:**
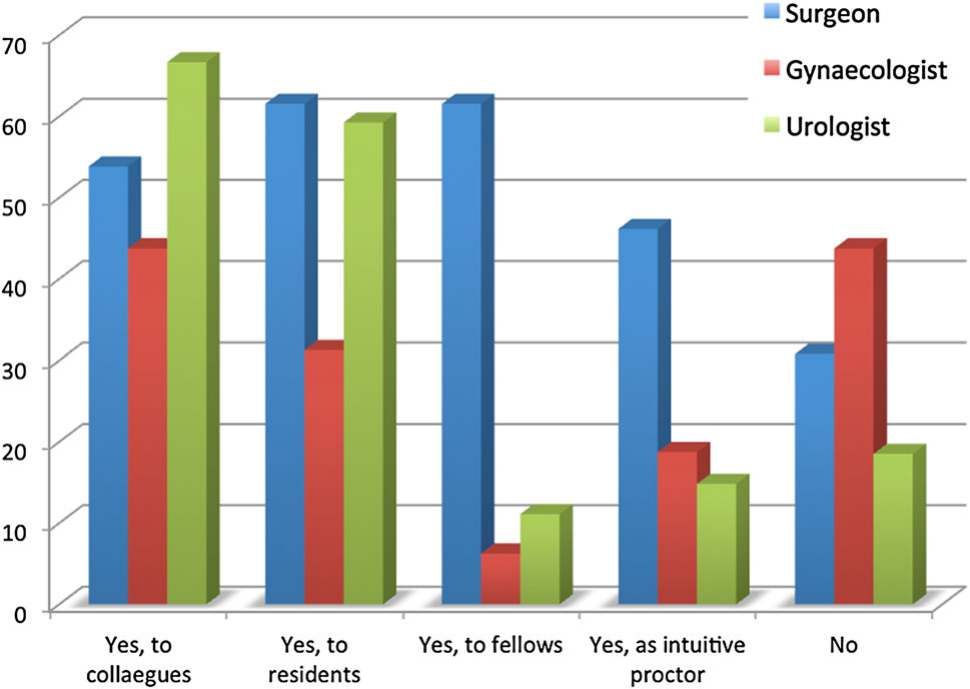
Do you teach robot-assisted surgery?

**Fig. 5:**
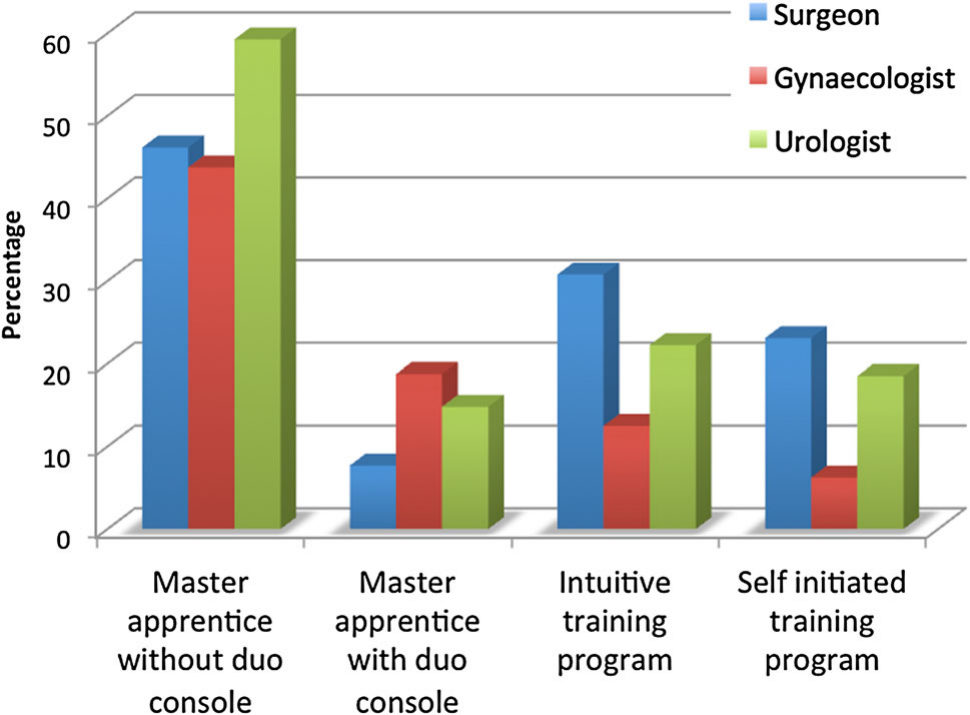
How do you teach robot-assisted surgery?

As for teaching robotic surgical skills, more than half of the respondents (71.6 %) do so to colleagues, residents, and fellows (Fig. 4). This did not differ significantly among the surgical specialties (Pearson Chi-square test: 3.174; *p* value = 0.205). Of the teaching respondents, 32.5 % claimed to be a proctor for Intuitive Surgical. The robotic skills education varied from master-apprenticeship with or without duo console to a structured training program created by Intuitive Surgical and a self-designed training curriculum (Fig. 5).

### Opinion on multidisciplinary basic skills training

**Fig. 6:**
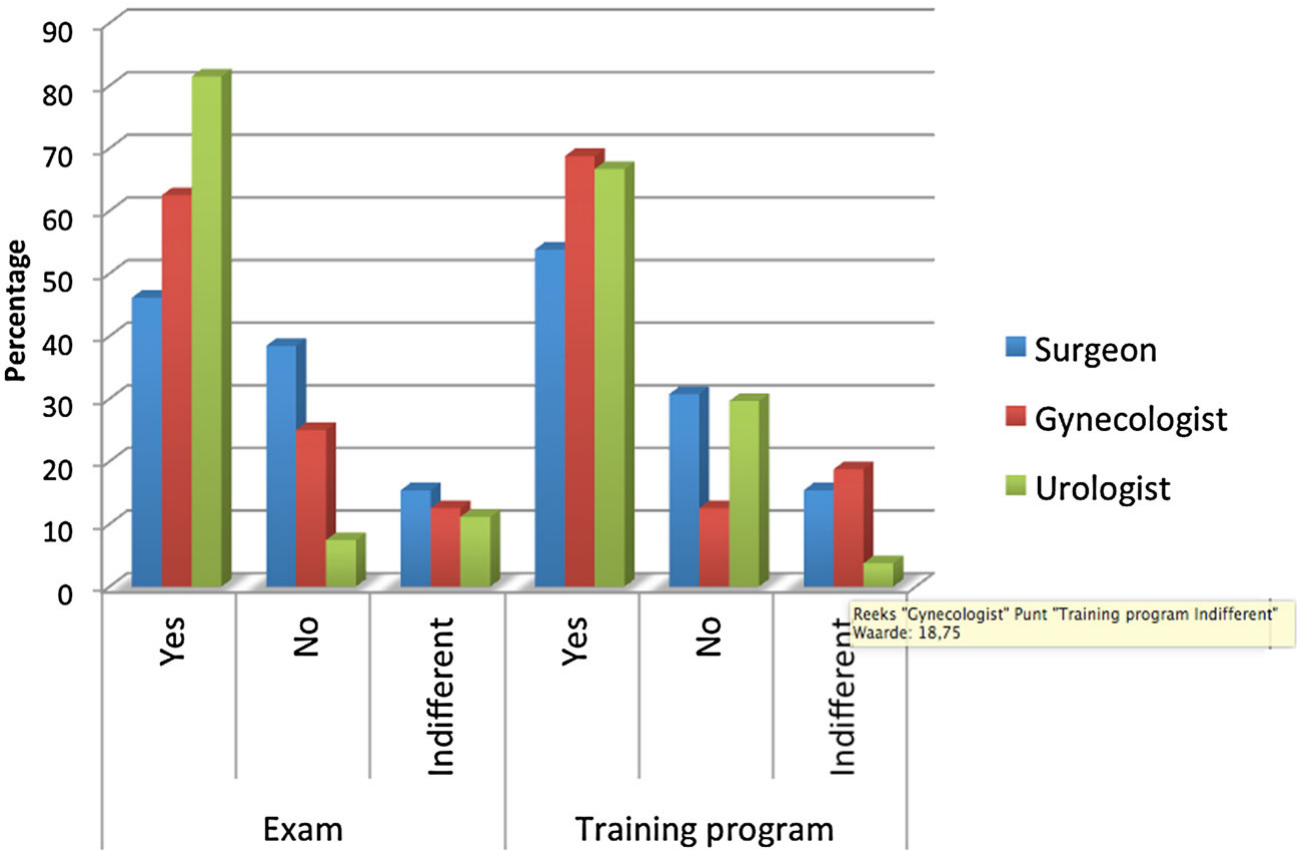
Do you agree on setting a structured and obliged training program for basic robotic skills? And do you think an examination or test should be installed before starting robot-assisted surgery?

The respondents were asked whether they agreed on setting a structured and obliged training program for basic robotic skills: 65 % agreed, 25 % disagreed, and 10 % had no opinion. To the question, do you think an exam or test should be installed before starting robot-assisted surgery, 68 % of the respondents agreed, 19 % disagreed, and 12 % had no opinion (Figure 6).

## Discussion

With this study, we aimed to answer three research questions. The first two questions were: What are the training pathways followed by the current robot surgeons? and Are there differences between the specialties in robot training and robot use? The results of the questionnaire demonstrate that urologists use the robot significantly more often than the surgeons and gynecologists. This is not surprising since we know that the da Vinci robot has especially landed in urology [6]. The results of this questionnaire revealed a wide variation of training programs. The kind of training varied from a structured training program by Intuitive Surgical, master-apprenticeship with or without duo console, fellowship, and self-designed training programs. The number of hours differed among individuals, but did not show significant differences between the different specialties. Also, the year of start with robotic surgery did not significantly influence the type or amount of training. Although one should expect that training and especially supervision would increase over the years with an increasing availability of supervisors, our data do not show this. This is in line with previous research among European urologists [7]. Possibly, the heterogeneity among robot starters was too large to show differences. For instance, some ‘early’ starters were already the second operator in their hospital with an experienced colleague. Also, some ‘late’ starters were the first in their hospital without a more experienced colleague to teach them.

Remarkable is that the majority of respondents spent less than 5 h on basic skills training. Recent literature shows an average of 10 h of skills training is needed to reach proficiency in basic robotic skills [8]. Most likely, the basic skills training of the professionals was time based instead of criterion based. Recent literature data suggested that the optimal endpoint for simulator training is the attainment of a predefined level (criterion-based training), rather than the completion of an arbitrary number of procedures, task repetitions, or hours using the simulator (time-based training) [9–11]. It can be doubted that all robot professionals completed their learning curve in basic robotic skills within 5 h of training. A multidisciplinary basic skills training and examination based on clear criteria can assure a proper end of training level for all trainees and can prevent that the learning curve of basic skills is completed on patients.

Another finding is that almost all respondents had previous laparoscopic experience. Conventional laparoscopy was already quiet common in surgery, gynecology, and urology in The Netherlands. And most likely, those already interested in minimal invasive surgery started robot-assisted surgery. Therefore, most robot users had already previous experience in laparoscopy. New generations will most likely receive less laparoscopy training since the robot is taking a large share of minimal invasive procedures, especially in urology. This makes a basic training even more important since the principals of minimal invasive abdominal surgery, for instance working with the pneumoperitoneum, should be taught to a new generation.

We did not find any significant differences in robot training between the specialities. This is a positive finding and shows that all specialities put effort in basic training. This can be explained by the fact that the manufacturer offered all new robot costumers a mandatory introduction training to the da Vinci Surgical System, independent of their speciality. This training guaranteed a basic level for all robot users. Nevertheless, there is a wide range in training hours among all specialties since training after the introduction course was not mandatory and was self-initiated. For residents or fellows, it will be different since new users are not new customers, so the training program provided by the manufacturer is not mandatory for them.

As last research question, we asked the participants about their opinion on multidisciplinary basic skills training. The majority of the respondents favor an obliged training program and examination for the basics of robot skills training. As robotic surgery is expanding throughout various fields of surgery, there is still no consensus on a validated training curriculum. In 2010, the Dutch Health Care Inspectorate (IGZ) published a report ‘Insufficiently prepared introduction of robotic surgery’ [12]. This report noted insufficient criteria for the surgeon’s competence before starting with robotic surgery in 50 % of hospitals. Earlier, in 2007, a similar report was published about the risks of minimally invasive surgery that were being underestimated. Overall, these reports support the notion that training in endoscopic surgery needs to be improved and that a national multidisciplinary consensus is essential [13]. This confirms the increasing need for certified training and assessment criteria for residents, fellows, and surgeons. A structured multidisciplinary training program could be an effective starting point.

The majority of robot professionals in The Netherlands agree that a structured multidisciplinary training program should be implemented for the basics in robotic surgery. The time seems right since the majority of the professionals indicate that they teach residents robotic surgery and robotic skills acquirement is in particular improved at younger age. With the available literature on robot training [14, 15], a program can be developed with well-defined proficiency standards to safeguard the quality of care and prevent learning by doing directly on the patient.

A multidisciplinary training program could consist of items such as knowledge training, basic skills training, draping and docking, and patient positioning. Also, some general safety issues and anesthetic difficulties etcetera could be covered in a multidisciplinary training. In our opinion, these components could be trained and tested in no longer than 1- or 2-day course if participants come well prepared. After a broad criterion-based multidisciplinary basic training, a procedure and speciality-specific training could follow. Items that are more procedure specific such as video observations and table assisting are less suitable to cover multidisciplinary.

A limitation of our study is that the group studied was a selection (62 %) of all robot professionals. The participants were willing to complete a questionnaire about their training. Possibly, this led to a selection bias that influenced the reported opinions on training robotics. Another limitation is that we do not know the reason professionals would be against a training program. Before implementing a program, this would be very useful information to know.

## Conclusions

Training of the current robot professionals is mostly dependent on local circumstances and the manufacturer of the robot system. Training is independent of the year of start with robotic surgery and speciality. To guarantee the quality of future training of residents and fellows in robot-assisted surgery, clear training goals should be formulated and implemented. Since this study shows that current training of different specialities does not differ, training in robotic surgery could be started by a multidisciplinary basic skills training and assessment.

## Electronic supplementary material

Below is the link to the electronic supplementary material.

Supplementary material 1 (PDF 77 kb)
