# Supplementary material for: Current training on the basics of robotic surgery in the Netherlands: Time for a multidisciplinary approach?
Source: Surg Endosc. 2016 May 18;31(1):281–7. doi: 10.1007/s00464-016-4970-2 (PMC5216079; doi:10.1007/s00464-016-4970-2)
Supplement: Supplementary file 1 — Supplementary material 1 (PDF 77 kb) [file 464_2016_4970_MOESM1_ESM.pdf]

1. What is your name?

2. What is your profession?

3. What is your age?

4. What is your gender?

5. Do you perform Robot assisted laparoscopic procedures?

Yes/No

6. Did you perform Robot assisted laparoscopic procedures in the past?

Yes/No

7. When did you start with Robot assisted laparoscopy?

8. Number of robot-assisted procedures performed

9. How many Robot assisted procedures do you perform a month?

- □ 0-5 procedures
- □ 5-10 procedures
- □ 10-15 procedures
- □ 15-20 procedures
- □ 20-25 procedures
- □ 25-30 procedures
- □ 30-35 procedures
- □ >35 procedures

10. Do you have conventional laparoscopic experience?

Yes/No

11. Number of conventional laparoscopic procedures performed?

12. How where you trained in robotic surgery?

□By intuitive surgical with a proctor

□By my supervisor (master-apprentice learning)

□Fellowship

□Different …..

13. What kind of robot training did you perform and how much time did you spend on the different parts?

	<5 h	5-10 h	10-20 h	20-30 h	30-40 h	40-50 h	> 50 h
Knowledge training							
Video observations							
Live observations							
Table assisting							
Basic skills training							
Animal or cadaver training							
Team training							

14. Did you take an exam before you started patient related work on the Da Vinci Robot?

□Yes, knowledge exam

□Yes, Skills exam

□Yes, knowledge and skills

□No

15. During how many procedures were you supervised (proctored)?

16. Did you use a duo console?

Yes/No

17. Do you personally teach robot assisted laparoscopy?

(more answers possible)

□Yes, to colleagues

□Yes to residents

□Yes, to fellows

□Yes as intuitive proctor

□No

18. How do you teach robot assisted laparoscopy?

(more answers possible)

□ Master apprentice without duo console

□ Master apprentice with duo console

□ Intuitive surgical training program

□ Self initiated training program

□ Different, ….

19. Do you agree on setting a structured and obliged training program for basic robotic skills?

Yes/No/No opinion

20. Do you think an exam or test should be installed before starting robot-assisted surgery

Yes/No/No opinion

21. Do you have any other comment?
